# Paracrine regulation in mesenchymal stem cells: the role of Rap1

**DOI:** 10.1038/cddis.2015.285

**Published:** 2015-10-22

**Authors:** Y Zhang, Z Zhang, F Gao, H-F Tse, V Tergaonkar, Q Lian

**Affiliations:** 1 Department of Medicine, Cardiology Division, University of Hong Kong, Hong Kong SAR, China; 2 Department of Ophthalmology, Li Ka Shing Faculty of Medicine, University of Hong Kong, Hong Kong SAR, China; 3 Research Centre of Heart, Brain, Hormone, and Healthy Aging, Li Ka Shing Faculty of Medicine, University of Hong Kong, Hong Kong SAR, China; 4 Institute of Molecular and Cellular Biology, Biopolis, Buona Vista, Singapore

Since the first report more than a decade ago that bone marrow (BM) stem cells transplantation could promote heart regeneration following myocardial infarction (MI),^1^ different types of stem cells have been examined. Among those investigated to date, mesenchymal stem cells (MSCs) have attracted huge interest owing to some of their unique properties that include easy isolation, high expandability and low immunogenicity. Nonetheless despite the promising results of animal studies and clinical trials, MSC-based therapy for MI still faces some fundamental hurdles.^2^ One of the major challenges is the poor cell engraftment and low cell-survival rate following transplantation caused by the hostile environment of the injured heart and change to the immunologic milieu of transplanted MSCs. In addition, the mechanisms of stem cell-mediated tissue repair are not fully understood. Low cell retention and poor myocardial differentiation cannot explain the remarkable recovery of heart function following MSCs treatment. Therefore, the paracrine effects of MSCs are currently thought to have a predominant role. Although many cytokines released from MSCs contribute to heart function recovery,^3^ some are useless and even harmful.^4^ Thus, there is an urgent need to optimize MSCs before their transplantation to improve cell survival and augment their paracrine effects. Recently, Rap1, a telomeric repeat binding factor 2 interacting protein 1, has been identified as an important modulator of the nuclear factor kappa-B (NF-*κ*B) pathway.^5, 6^ This pathway has been reported to regulate MSCs secretion profiling and survival.^7, 8^ On the basis of these findings, modulation of the NF-*κ*B pathway to mediate MSCs therapy is feasible and important. Nonetheless total deletion of NF-*κ*B is lethal to cells.^9^ Identification of an important regulator that can mediate activity of the NF-*κ*B pathway and subsequently regulate MSC therapeutic efficacy for MI is vital. The roles of Rap1 in regulation of MSCs and the underlying mechanisms have not been classified, thus to understand how Rap1 regulates the paracrine effects and cell survival of MSC-mediated heart repair following infarction by regulation of the NF-*κ*B pathway, therefore, is important.^10^

In this study,^10^ wild-type BM-MSCs and Rap1^−/−^-BM-MSCs were derived from wild-type and Rap1^−/−^ litter mate mice. It appears that both wild type and Rap1 deficiency BM-MSCs share the same MSCs properties including cell-surface markers and multipotent-lineage differentiation potential. Fluorescence-activated cell sorting confirmed that BM-MSCs and Rap1^−/−^-BM-MSCs express Sca-1, CD90, CD105, but not CD45 or CD34. Moreover, wild-type BM-MSCs and Rap1^−/−^-BM-MSCs can differentiate into adipocytes, chondrocytes and osteocytes. The relationship of Rap1 and NF-*κ*B was examined. NF-*κ*B activity of stromal cells was increased by Rap1, as measured by pNF-*κ*B-luciferase activity reporter activity, and abolished by IkB dominant negative protein. Knock down of Rap1 with shRap1 resulted in diminished translocation of NF-*κ*B-p65 from the cytoplasm to the nuclei in response to TNF-*α*-stimulation. The cell survival of Rap1^−/−^-BM-MSCs and wild-type BM-MSCs under normoxic or hypoxic condition was investigated. There was no significant difference in apoptosis of Rap1^−/−^-BM-MSCs and wild-type BM-MSCs under normoxic conditions. Nonetheless under hypoxic conditions, the apoptotic rate of Rap1^−/−^-BM-MSCs was much lower than that of wild-type BM-MSCs. This suggests that Rap1^−/−^-BM-MSCs are more tolerant than wild-type BM-MSCs to hypoxia-induced apoptosis. Meanwhile, Rap1^−/−^-BM-MSCs displayed a significantly reduced ratio of phosphorylated NF-*κ*B-p65 to NF-*κ*B-p65 and ratio of Bax to Bcl-2 compared with wild-type BM-MSCs, indicating that the absence of Rap1 enhances the resistance of MSCs to the hypoxic challenge through regulation of NF-*κ*B activity. The resistance of Rap1^−/−^-BM-MSCs to apoptosis was reduced when Rap1 was overexpressed in Rap1^−/−^-BM-MSCs. Moreover, compared with wild-type BM-MSCs, pro-inflammatory paracrine cytokines including TNF-*α*, IL-6 and MCP-1 were greatly reduced in Rap1^−/−^-BM-MSCs in a hypoxic environment. The cardioprotective effects of hypoxic-conditioned medium (CdM) of Rap1^−/−^-BM-MSCs and wild-type BM-MSCs were also tested. The apoptosis of neonatal cardiomyocytes induced by hypoxia was significantly reduced when co-cultured with Rap1^−/−^-BM-MSCs hypoxic-CdM compared with wild-type BM-MSCs hypoxic CdM. The increased cardioprotective effects of Rap1^−/−^-BM-MSCs hypoxic CdM were reduced when Rap1^−/−^-BM-MSCs were reconstituted with Rap1 re-expression. To further examine the therapeutic effects of Rap1^−/−^-BM-MSCs in MI, Rap1^−/−^-BM-MSCs and wild-type BM-MSCs were injected into a mouse model of MI. Results revealed that 4 weeks following transplantation, the cell-survival rate in ischemic heart tissue was much higher for Rap1^−/−^-BM-MSCs than wild-type BM-MSCs but there was no significant difference in differentiation into cardiomyocytes. Compared with wild-type BM-MSCs, transplantation of Rap1^−/−^-BM-MSCs also significantly improved heart function, prevented heart remodeling and reduced cardiomyocyte apoptosis. In addition, transplantation of Rap1^−/−^-BM-MSCs greatly reduced the inflammatory response in the ischemic heart compared with BM-MSCs.

This study has several important findings (Figure 1). First, the absence of Rap1 of MSCs strongly regulates secretion profiling and especially reduces the release of pro-inflammatory cytokines and enhances resistance to the stressful challenge. Second, these paracrine effects are attributed to the regulation of the NF-*κ*B signal pathway by Rap1. Third, transplantation of Rap1^−/−^-BM-MSCs greatly improves heart function recovery following MI is associated with reduced inflammation and enhanced cell survival. Therefore, selective inhibition of Rap1 in BM-MSCs presents a novel strategy to enhance future MSC-based therapy by regulating paracrine profiling.

## Figures and Tables

**Figure 1:**
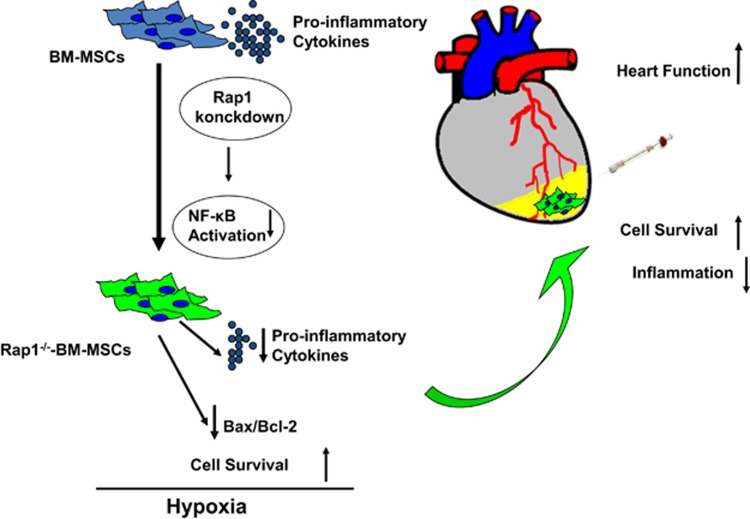
Transplantation of Rap1^−/−^-BM-MSCs greatly improved heart function recovery post MI through reduction of inflammation and enhancement of cell survival that is regulated by NF-*κ*B signal pathway
